# Use of ultrasound imaging Omics in predicting molecular typing and assessing the risk of postoperative recurrence in breast cancer

**DOI:** 10.1186/s12905-024-03231-8

**Published:** 2024-07-02

**Authors:** Xinyu Song, Haoyi Xu, Xiaoli Wang, Wen Liu, Xiaoling Leng, Yue Hu, Zhimin Luo, Yanyan Chen, Chao Dong, Binlin Ma

**Affiliations:** 1 Department of Breast and Thyroid Surgery, Tumor Hospital Affiliated to Xinjiang Medical University, No. 789 of Suzhou Street, Xinshi District, Urumqi, 830000 China; 2 Department of Artificial Intelligence and Smart Mining Engineering Technology Center, Xinjiang Institute of Engineering, Urumqi, 830023 China; 3 Department of Ultrasound, The Tenth Affiliated Hospital of Southern Medical University, Dongguan, 523000 China; 4 Department of Breast Cancer Center Diagnosis Specialist, Sun Yat-sen Memorial Hospital, Guangzhou, 510120 China; 5 Department of General Surgery, Tori County People’s Hospital, Tuoli, 834500 China

**Keywords:** Breast cancer, Molecular typing, Postoperative recurrence risk, Prediction, Ultrasound imaging omics

## Abstract

**Background:**

The aim of this study is to assess the efficacy of a multiparametric ultrasound imaging omics model in predicting the risk of postoperative recurrence and molecular typing of breast cancer.

**Methods:**

A retrospective analysis was conducted on 534 female patients diagnosed with breast cancer through preoperative ultrasonography and pathology, from January 2018 to June 2023 at the Affiliated Cancer Hospital of Xinjiang Medical University. Univariate analysis and multifactorial logistic regression modeling were used to identify independent risk factors associated with clinical characteristics. The PyRadiomics package was used to delineate the region of interest in selected ultrasound images and extract radiomic features. Subsequently, radiomic scores were established through Least Absolute Shrinkage and Selection Operator (LASSO) regression and Support Vector Machine (SVM) methods. The predictive performance of the model was assessed using the receiver operating characteristic (ROC) curve, and the area under the curve (AUC) was calculated. Evaluation of diagnostic efficacy and clinical practicability was conducted through calibration curves and decision curves.

**Results:**

In the training set, the AUC values for the postoperative recurrence risk prediction model were 0.9489, and for the validation set, they were 0.8491. Regarding the molecular typing prediction model, the AUC values in the training set and validation set were 0.93 and 0.92 for the HER-2 overexpression phenotype, 0.94 and 0.74 for the TNBC phenotype, 1.00 and 0.97 for the luminal A phenotype, and 1.00 and 0.89 for the luminal B phenotype, respectively. Based on a comprehensive analysis of calibration and decision curves, it was established that the model exhibits strong predictive performance and clinical practicability.

**Conclusion:**

The use of multiparametric ultrasound imaging omics proves to be of significant value in predicting both the risk of postoperative recurrence and molecular typing in breast cancer. This non-invasive approach offers crucial guidance for the diagnosis and treatment of the condition.

## Background

In recent years, the incidence of breast cancer (BC) has witnessed a consistent rise, surpassing lung cancer to emerge as the foremost malignant tumor affecting women globally [1]. Notably, it stands as the leading cause of mortality among women worldwide. Statistical data indicate that the prevalence of breast cancer among young women in China exceeds that of other nations, presenting a substantial threat to the physical and mental well-being of Chinese women [2]. A molecular typing-based classification system was introduced at the St. Gallen conference in 2013, categorizing breast cancer into four subtypes: luminal A, luminal B, Human Epidermal Growth Factor Receptor 2 (HER-2) overexpression, and Triple Negative Breast Cancer (TNBC). Currently, the primary modalities used in breast cancer treatment encompass surgery, targeted therapy, endocrine therapy, chemotherapy, and radiotherapy [3, 4]. For patients exhibiting positive estrogen receptor (ER) or progestogen receptor (PR), supplementary endocrine therapy is recommended to manage tumor progression and enhance prognosis [5]. Moreover, patients with HER-2 overexpression may undergo additional targeted therapy [6]. TNBC, characterized by the lack of ER, PR, and HER-2 expression [7], exhibits limited responsiveness to endocrine and targeted therapies, necessitating standardized chemotherapy as a primary therapeutic approach alongside surgical interventions [8, 9]. 

To enhance the prognosis of patients diagnosed with breast cancer, the pivotal focus lies in early diagnosis and timely intervention. Molecular typing of breast cancer and the assessment of postoperative recurrence risk are crucial factors, enabling clinicians to formulate personalized treatment strategies and evaluate patient prognoses [10–13]. Guidelines established by the Chinese Society of Clinical Oncology (CSCO) offer appropriate regimens based on factors such as the number of lymph node metastases, molecular typing, histological grading, and tumor size. Treatment modalities, incorporating anthracyclines, paclitaxel, cyclophosphamide, and platinum, are further supplemented with targeted therapies or endocrine therapies based on the assessed risk of recurrence, thereby providing patients with individualized and precise treatment plans. The China Anti-Cancer Association (CACA) guidelines categorize postoperative recurrence risk as high, intermediate, or low, with a focus on investigating intermediate- and high-risk patient groups, given the scarcity of low-risk cases in clinical practice. Crucial to enhancing patient prognosis and quality of life, current preoperative diagnostic techniques for breast cancer predominantly encompass mammography, ultrasound, and magnetic resonance imaging (MRI) [10, 14]. However, the high proportion of dense mammary gland tissue among Chinese women with breast cancer contributes to a notably high false-positive rate in X-ray-based screening, ranging from 65 to 90% [15, 16]. While MRI is characterized by its accuracy, it is cost-prohibitive and time-consuming. Ultrasonography, a painless, non-invasive, cost-effective, and expeditious method, surpasses mammography and MRI in terms of detection rate, accuracy, and cost-benefit ratio among Chinese women, emerging as the primary screening modality for breast diseases [17]. Currently, breast cancer molecular typing and postoperative histopathology results are typically derived from preoperative puncture or postoperative pathology of immunohistochemistry. However, clinical observations reveal differences between core needle punctures of mammary glands and immunohistochemistry of surgical specimens, potentially leading to increased risks of recurrence, metastatic recurrence, and mortality [18]. This discrepancy may stem from variations in immunohistochemistry results within different locations of the same cancer focus, exhibiting differing proportions. Studies indicate that receptor status may undergo changes after neoadjuvant treatment, showcasing inconsistencies of approximately 3–5% in hormone receptor (HR) status and 10% in HER-2 status in breast cancers treated with current neoadjuvant regimens [19]. Studies emphasize the prognostic implications of changes in immunohistochemistry post-treatment, recommending the retesting of biomarkers following neoadjuvant treatment or upon the development of drug resistance [20, 21]. Such reevaluation aims to tailor treatment regimens, mitigate the risk of postoperative recurrence, and enhance patient prognosis. Presently, immunohistochemistry relies on clinical specimens. However, it is susceptible to variations based on site selection and sectioning levels, leading to somewhat inaccurate results and a considerable wait time. Rapid and accurate prediction of the molecular typing and postoperative recurrence risk among patients during the disease course could serve to prompt clinicians on the necessity of updating immunohistochemistry results, potentially extending patient survival and enhancing overall quality of life.

With technological advancements, there is a growing inclination toward multimodal imaging. Multimodal imageomics technology facilitates the extraction of numerous image features from existing medical images in a high-throughput manner. Automated data characterization algorithms are then applied to transform the image data from the region of interest (ROI) into high-resolution feature data. This data can be effectively explored to construct clinical prediction models, providing more comprehensive and supplementary information for the diagnosis and treatment of diseases [22, 23]. The role of multimodal imageomics technology in the auxiliary diagnosis and treatment of diseases has been widely studied, including CT, MRI, ultrasound images, etc. Clinical prediction models based on multimodal imageomics techniques have shown great potential in the diagnosis of diseases [24]. Different imageomics techniques are suitable for different diseases, for example, CT radiomics and deep learning based models perform well in staging lymph node metastasis in pancreatic cancer [25], and for neurological diseases, MRI imageomics and deep learning models have greater potential. In one study, its combined accuracy in distinguishing between neuromyelitis optica spectrum disorders and multiple sclerosis was 82% [26], and deep learning-based ultrasound imageomics is more suitable for breast tumor-related differentiation and diagnosis. Notably, a study demonstrated that a deep learning model in breast cancer diagnosis achieved a classification accuracy of 97.18% in distinguishing malignant, benign, and normal ultrasound images [27]. Another study highlighted the efficacy of multiparametric ultrasound imaging omics in predicting molecular subtypes of breast cancer, with an area under the curve (AUC) of 0.970 for the prediction of triple-negative and non-triple-negative breast cancers [28]. Research data have shown that in the accurate diagnosis of breast cancer, the accuracy of deep learning model in diagnosing malignant tumours in BI-RADS 4a patients is 92.86%, which theoretically reduces unnecessary biopsies by 67.86% [29], increasing diagnosticity while significantly reducing invasive operations for patients. In another study using a deep learning model of ultrasound images to discriminate breast fibroadenomas from lobular breast tumors, the AUC value reached 0.91 [30]. Therefore, the combined application of multimodal ultrasound technology has a broad application prospect for the diagnosis and prognosis of breast cancer.

However, since there is still no in-depth research on multimodal ultrasound technology in determining the risk of postoperative recurrence of breast cancer and the four molecular subtypes, the present study is intended to establish a model by extracting the characteristics of ultrasound images of patients with different types of subtypes and different risks of postoperative recurrence to predict the molecular subtypes and the risk of postoperative recurrence in patients with breast cancer, which is aimed at providing an effective guide to the diagnosis and treatment of breast cancer in a non-invasive way.

## Materials and methods

### Study participants

Between January 2018 and June 2023, we conducted a retrospective study encompassing 534 cases of female patients diagnosed with breast cancer through surgical procedures at the Affiliated Cancer Hospital of Xinjiang Medical University. The inclusion criteria encompassed the following: (1) Surgical pathological diagnosis in our hospital; (2) breast and axillary ultrasound examination performed in our hospital 15 days before surgery with clear and recognizable lesions; (3) complete clinical, pathological, and ultrasound data; (4) absence of preoperative endocrine, radiotherapy, or chemotherapy treatment; (5) no history of breast cancer in the patients and their relatives; (6) signed informed consent. The exclusion criteria comprised: (1) Male breast cancer was ruled out due to the lower number of male breast cancers and the difference in hormone levels compared to females; (2) Preoperative neoadjuvant therapy results in changes in receptor expression and ultrasound image characteristics, so it is excluded; (3) Previous breast cancer or other malignancies may affect breast cancer pathology and ultrasound image characteristics due to treatment or changes in the body’s immune microenvironment; patients with a history of previous cancer were excluded from this study; (4) To minimize bias, clinical and pathological data and ultrasound images were excluded if any of them were missing; (5) The number of patients with a low risk of postoperative recurrence is small, and to avoid imbalance in the data, only patients with an intermediate and high risk of postoperative recurrence were studied in this study.

### Clinical data collection

Data on clinical features of patients with breast cancer were retrospectively collected from the follow-up and medical record systems of our hospital. This information encompassed age, gender, ethnicity, pathological features (lesion size, histological grading, vascular tumor embolus, ER expression, PR expression, HER-2 expression, nerve invasion, and axillary lymph node metastasis), ultrasonographic features (aspect ratio, morphology, margins, posterior echogenicity, intra-lesional blood flow in the lesion, internal echoes, presence or absence of calcification, and lymph node morphology), tumor TNM (Tumor Node Metastasis) clinical staging, molecular typing, and the risk of postoperative recurrence. Patients were categorized into groups based on the latest CSCO guidelines for clinical molecular typing of breast cancer: luminal A group, luminal B group, HER-2 overexpression group, and TNBC group [31]. Furthermore, patients were classified into intermediate-risk and high-risk groups based on the risk of postoperative recurrence using the latest criteria from the CACA guidelines [32]. 

### Instruments and methods

#### Ultrasound image acquisition

Breast ultrasound image acquisition was conducted by an experienced radiologist, who was blinded to the pathological results. A GE Logic E9 color Doppler ultrasound machine, equipped with a line-array probe, was used for the procedure. The patient assumed the supine position with arms abducted by 90° to fully expose the mammary glands and axilla. Radial scanning initiated clockwise from the outer upper quadrant, centered on the nipple, with overlapping adjacent areas scanned. Ultrasound characteristics of the breast mass and axillary lymph node metastasis were collected from transverse, longitudinal, and radial scanning views, with eligibility criteria requiring the presence of clear and interpretable two-dimensional views.

#### Radiomics feature extraction and analysis

To enhance the efficiency and precision of outlining the ROI, a concurrent application of manual outlining and artificial intelligence outlining was used. The manual outlining, conducted in a double-blind manner, was executed by a senior radiologist with 10 to 15 years of experience. This radiologist outlined the ROIs and labeled them for storage. The radiologist always uses the same ultrasound machine for image acquisition, avoiding squeezing the tumor as much as possible during the process. The maximum transverse diameter and the maximum longitudinal diameter of the tumor are captured separately, and at least two clear images are saved. The acquired images avoided blood vessels, nerves and ribs as much as possible to minimize the interference with the images and maximize the quality of the images. Unet software was used for the AI outlining segment. In order to test the accuracy of Unet software, we randomly selected 100 ultrasound images, numbered 1-100, and duplicated the copies, one of which outlined the region of interest (ROI) using Unet software, and the other manually outlined the ROI. The ROI was cut and then the overlap of the two images with the same number was compared using the Unet software, resulting in Intersection over Union (IoU) = 0.973, suggesting that the Unet method is accurate. Following the image outlining process, the images were input into the “Pyradiomics” feature package (github.com/Radiomics/pyradiomics) for feature extraction. A total of 744 features were extracted, encompassing shape parameters, first-order parameters, gray-level co-occurrence matrix parameters (GLCM), gray-level run-length matrix (GLRLM) parameters, gray-level size zone matrix (GLSZM) parameters, and gray-level dependence matrix (GLDM) parameters. To address errors arising from inconsistent sample sizes across classifications, the Synthetic Minority Oversampling Technique (SMOTE) was used. SMOTE algorithm is a classic method to solve unbalanced dataset, its full name is Synthetic Minority Over-sampling Technique. SMOTE algorithm is based on the principle of balancing the dataset by synthesizing new minority samples to improve the model performance. It creates new synthetic samples by interpolating between the minority class samples to balance the dataset. The core idea of the SMOTE algorithm is based on the K-nearest neighbor algorithm. For each minority class sample, SMOTE calculates its K nearest neighbor samples and then generates a new sample between two randomly selected nearest neighbors. The image features were divided into a training set and a validation set in a 7:3 ratio, and data normalization was carried out to transform all features between − 1 and 1 using maximum absolute normalization. The Intra-class Correlation Coefficient (ICC) was calculated to retain features with an ICC > 0.75. The LASSO regression was then applied for multiple dimensionality reduction of the data. Finally, features with significant predictive value for both the molecular typing of breast cancer and the risk of postoperative recurrence were identified.

#### Model construction

The SVM algorithm was used to construct predictive models using the specific features identified through the LASSO method. In the SVM algorithm, the value of test_size is set to 0.3, the kernel function is set to rbf, and the gamma value is set to scale. In the LASSO regression analysis, the specific parameters we set at runtime are: the value of test_size is 0.3, the value of random_state is 15, the value of n_estimators is 200, the value of random_state_rf is 20, the criterion is set to entropy, the class_weight is set to balanced, and the Lasso Alpha parameter is set to scale. weight is set to balanced, Lasso Alpha parameter is -4, 1, 50, the number of iterations Lasso max_iter is 100,000, and lasso is set to tenfold cross-validation. Subsequently, the receiver operating characteristic (ROC) curve for the histological model was generated. To evaluate the consistency of the predictive model with the ideal model, a calibration curve was employed. Furthermore, the clinical practicability of the model was assessed using the decision curve.

### Statistical analysis

The data underwent analysis using SPSS 26.0 software, and the SMOTE algorithm was used to address sample size imbalances within each subgroup. For measurement data, the normality of distribution was initially assessed through the Kolmogorov–Smirnov test. Normally distributed data are presented as mean ± standard deviation (x̅±s), and the independent samples *t*-test was applied for comparisons. Non-normally distributed data are expressed as median (upper quartile, lower quartile) and analyzed using the Mann–Whitney U test. Count data are presented as frequencies, and the chi-squared test and Fisher’s test were used to verify data distribution. A multifactor logistic regression model was constructed to identify relevant influencing factors affecting the molecular typing of breast cancer and the risk of postoperative recurrence. Python 3.6 and Matplotlib software were used to generate the ROC curve, calibration curve, and decision curve. The AUC, sensitivity, specificity, and accuracy served as evaluation indicators for the model performance. Statistical significance was considered when the p-value was less than 0.05.

## Results

### Comparison of baseline data of clinical information

In this study, 534 cases were ultimately enrolled, comprising 311 cases classified as having an intermediate risk of postoperative recurrence and 223 cases classified as having a high risk of recurrence. Statistical analysis revealed significant differences (*P* < 0.05) among female patients diagnosed with breast cancer having distinct postoperative recurrence risks in the following indicators: the number of lymph node metastases, lesion size, histological grading, vascular tumor embolus, nerve invasion, ER expression, PR expression, HER-2 expression, proliferation marker (Ki-67) expression, molecular typing, clinical staging, and ultrasound image characteristics (blood flow, mass morphology, mass margins, lymph node morphology, internal calcification) (refer to Table 1). Among the enrolled cases, there were 87 cases of luminal A, 234 cases of luminal B, 84 cases of HER-2 overexpression, and 129 cases of triple-negative breast cancer. Upon analyzing the clinical data and ultrasound characteristics, statistically significant differences (*P* < 0.05) were observed among the four groups of molecular typing in female patients diagnosed with breast cancer in the following indicators: ethnicity, number of lymph node metastases, lesion size, histologic grading, expression of Ki-67, risk of postoperative recurrence, clinical stage, and features of ultrasound images (mass morphology, internal echogenicity, abnormal lymph node morphology, and internal calcification) (refer to Table 2).

**Table 1:** Comparison of baseline data of different postoperative recurrence risk groups

Baseline parameters	Intermediate risk, *n* = 311	High risk, *n* = 223	Statistic (Z, χ^2^)	*P* value*
Age (years) 0.50 (0.25–0.75)	51.00 (45.00 ~ 59.00)	52.00 (45.00 ~ 58.00)	-0.559	0.576
Ethnicity (number)				
Ethnic Han	192	124	2.02	0.155
National minority	119	99		
Axillary lymph node metastasis (number) 0.50 (0.25–0.75)	0.00 (0.00 ~ 0.00)	5.00 (2.00 ~ 9.00)	-18.275	< 0.001
Size of lesion (cm) 0.50 (0.25–0.75)	2.00 (1.50 ~ 2.80)	3.00 (2.50 ~ 4.55)	-4.12	< 0.001
Histologic grading				
I	5	1	9.598	0.008
II	83	37		
III	223	185		
Vascular tumor embolus (number)				
Yes	105	155	66.42	< 0.001
No	206	68		
ER expression (%) 0.50(0.25 ~ 0.75)	90.00 (5.00 ~ 90.00)	30.00 (0.00 ~ 90.00)	-5.031	< 0.001
PR expression (%) 0.50(0.25 ~ 0.75)	60.00 (0.00 ~ 80.00)	0.00 (0.00 ~ 70.00)	-5.851	< 0.001
HER expression (number)				
-/1+/2 + not amplified	289	161	42.10	< 0.001
2 + amplified/3+	22	62		
Nerve invasion (number)				
Yes	31	280	5.18	0.016
No	38	185		
Menopause (number)				
Yes	158	111	0.64	0.424
No	183	112		
Ki-67 (%) 0.50(0.25 ~ 0.75)	30.00 (20.00 ~ 50.00)	40.00 (30.00 ~ 70.00)	-5.374	< 0.001
Molecular typing (number)				
TNBC	62	62	62.82	< 0.001
Luminal A type	66	21		
Luminal B type	161	73		
HER-2 overexpression groups	22	62		
Clinical staging (number)				
I	124	2	277.79	< 0.001
II	174	67		
III	9	116		
IV	4	38		
Aspect ratio (number)				
> 1	173	135	1.28	0.257
< 1	138	88		
Posterior echo (number)				
Enhanced/Unchanged	147	90	2.511	0.113
Mixed/Attenuated	164	133		
Blood flow (number)				
Abundant	161	151	13.59	< 0.001
Little	150	72		
Morphology (number)				
Regular	25	7	5.53	0.019
Irregular	286	216		
Edge (number)				
Regular	6	4	0.013	1.000
Irregular	305	219		
Internal echo (number)				
Uniformly	7	4	0.13	0.769
Uneven	304	219		
Lymph node morphology (number)				
Normal	194	43	97.72	< 0.001
Abnormal	117	180		
Internal calcification (number)				
No	41	14	6.70	0.010
Normal	270	209		

Note: The first column on the left shows the name of clinical and ultrasound features; the second and third column shows the distribution of each clinical feature in the middle and high risk group; the fourth column is the statistical value; and the fifth column is the *P*-value. “ER” represents estrogen receptor, “PR” represents progestogen receptor, “HER” represents human epidermal growth factor receptor, “TNBC” represents triple-negative breast cancer, *Statistical significance was determined at *P* < 0.05

**Table 2:** Comparison of baseline data of different molecular typing groups

	TNBC *n* = 129	Luminal A type *n* = 87	Luminal B type *n* = 234	HER-2 overexpression *n* = 84	Statistic (Z, χ^2^)	*P*-value*
Age (years) 0.50(0.25 ~ 0.75)	51.00 (45.00 ~ 57.00)	50.00 (45.00 ~ 60)	51.00 (44.00 ~ 59.00)	52.00 (47.00 ~ 58.00)	1.37	0.712
Ethnicity (number)						
Ethnic Han	66	58	134	58	5.11	0.027
National minority	63	29	100	26		
Axillary lymph node metastasis (number) 0.50(0.25 ~ 0.75)	0.00 (0.00 ~ 2.00)	0.00 (0.00 ~ 3.00)	1.00 (0.00 ~ 4.00)	2.00 (0.00 ~ 5.00)	16.48	0.001
Size of lesion (cm) 0.50(0.25 ~ 0.75)	2.50 (1.95 ~ 3.10)	2.00 (1.50 ~ 3.00)	2.50 (1.80 ~ 3.63)	3.00 (2.25 ~ 4.28)	20.90	< 0.001
Histologic grading (number)						
I	1	3	2	0	73.52	< 0.001
II	28	45	46	1		
III	100	87	186	83		
Vascular tumor embolus (number)						
Yes	64	34	114	48	5.66	0.129
No	65	53	120	36		
Nerve invasion (number)						
Yes	16	15	22	16	6.85	0.077
No	113	72	212	68		
Menopause (number)						
Yes	64	37	129	48	3.79	0.285
No	65	50	114	36		
Ki-67 (%) 0.50(0.25 ~ 0.75)	60.00 (40.00 ~ 80.00)	10.00 (5.00 ~ 10.00)	30.00 (20.00 ~ 50.00)	50.00 (30.00 ~ 70.00)	253.70	< 0.001
Postoperative recurrence risk (number)						
Intermediate risk	62	66	161	22	62.92	< 0.001
High risk	67	21	73	62		
Clinical staging (number)						
I	30	31	55	10	27.03	0.001
II	70	32	103	36		
III	24	21	53	27		
IV	5	3	23	11		
Aspect ratio (number)						
> 1	78	50	130	50	0.96	0.811
< 1	51	37	104	34		
Posterior echo (number)						
Enhanced/Unchanged	63	34	104	36	2.11	0.550
Mixed/Attenuated	66	53	130	48		
Blood flow (number)						
Abundant	85	50	125	52	5.83	0.120
Little	44	37	109	32		
Morphology (number)						
Regular	121	86	211	84	15.10	0.002
Irregular	8	1	23	0		
Edge (number)						
Regular	125	84	231	84	4.28	0.233
Irregular	4	3	3	0		
Internal echo (number)						
Uniformly	126	82	231	84	8.38	0.039
Uneven	3	5	3	0		
Lymph node morphology (number)						
Normal	52	40	126	19	25.56	< 0.001
Abnormal	77	47	108	65	110.27 < 0.001	
Internal calcification (number)						
No	15	9	31	0	12.09	0.007
Normal	114	78	203	84	0.007	

Note: The first column on the left is the name and grouping of the clinical and ultrasound features; the second to fifth columns are the distribution of the clinical and ultrasound features in the four molecular typing groups. The sixth column is statistical value; the seventh column is *P*-value. “TNBC” represents triple-negative breast cancer, “HER” represents human epidermal growth factor receptor, * statistical significance was determined at *P* < 0.05

### Analysis of clinical features

The 22 clinical features underwent statistical analysis, resulting in the identification of 16 risk factors associated with the risk of postoperative recurrence through univariate analysis. Subsequently, these factors underwent multifactorial logistic regression analysis, ultimately revealing 6 independent risk factors: the number of lymph node metastases, ER expression, HER-2 expression, molecular typing, clinical staging, and ultrasonographic blood flow grading (refer to Table 3).

**Table 3:** Multiple logistic regression model analysis of the risk factors of postoperative recurrence

Subgroups	B	SD	Z	*P* value
Constant	-14.147	8.537	2.746	0.097
Number of axillary metastases	2.332	0.333	48.944	< 0.001
Lesion size	-0.086	0.192	0.201	0.654
Histologic grading	0.046	0.582	0.006	0.937
Vascular tumor embolus	-0.316	0.453	0.487	0.485
Nerve invasion	-0.399	0.579	0.474	0.491
ER expression	-0.034	0.012	8.336	0.004
PR expression	-0.001	0.01	0.002	0.96
HER-2 expression	4.603	1.018	20.444	< 0.001
Ki-67	0.015	0.011	1.805	0.179
Molecular typing	-1.077	0.364	8.78	0.003
Clinical staging	1.79	0.48	13.914	< 0.001
Morphology	0.201	1.176	0.029	0.864
Edge	1.788	3.603	0.246	0.62
Calcification	0.347	0.822	0.179	0.672
Lymph node morphology	-0.3	0.491	0.373	0.541
Blood flow grading	1.153	0.475	5.892	0.015

Note: “ER” represents estrogen receptor, “PR” represents progestogen receptor, “HER” represents human epidermal growth factor receptor, “B” represents the intercept, “SD” represents the slope, and “Z” represents the statistical value, and *P* < 0.05 is statistically significant. The results showed that the differences of number of axillary metastases, ER expression, HER-2 expression, molecular typing, clinical staging and ultrasonographic blood flow grading were statistically significant in the intermediate-risk and high-risk groups of recurrence risk

Using pathology as the gold standard, univariate analysis identified 11 risk factors associated with the molecular typing of breast cancer. Through multifactor logistic regression analysis of these 11 risk factors in the training set, 6 independent risk factors were discerned: Ki-67 expression, number of lymph node metastases, histological grade, postoperative recurrence risk, clinical staging, and lymph node morphology (refer to Table 4).

**Table 4:** Molecular typing model analysis using multivariate logistic regression

	x^2^	ν	*P* value
Intercept	0	0	
Lesion size	0.493	3	0.92
Ki-67	337.404	3	< 0.001
Ethnicity	7.155	3	0.067
Axillary lymph node metastasis (number)	41.055	3	< 0.001
Histologic grading	29.372	6	< 0.001
Postoperative recurrence risk assessment	106.015	3	< 0.001
Clinical staging	50.439	9	< 0.001
Morphology	1.688	3	0.64
Internal echo	4.294	3	0.231
Lymph node morphology	9.345	3	0.025
Calcification	6.297	3	0.098

Note: The first column on the left side is the name of clinical and ultrasound features, the second column is the chi-square value, the third column is the degree of freedom, and the fourth column is the p-value. The results showed that the Ki-67 expression, axillary lymph node metastasis number, histologic grading, postoperative recurrence risk assessment, clinical staging and lymph node morpholog in the four molecular typing groups was statistically significant

### Selection of radiomics features and model construction

#### Results of screening radiomics features

**Fig. 1:**
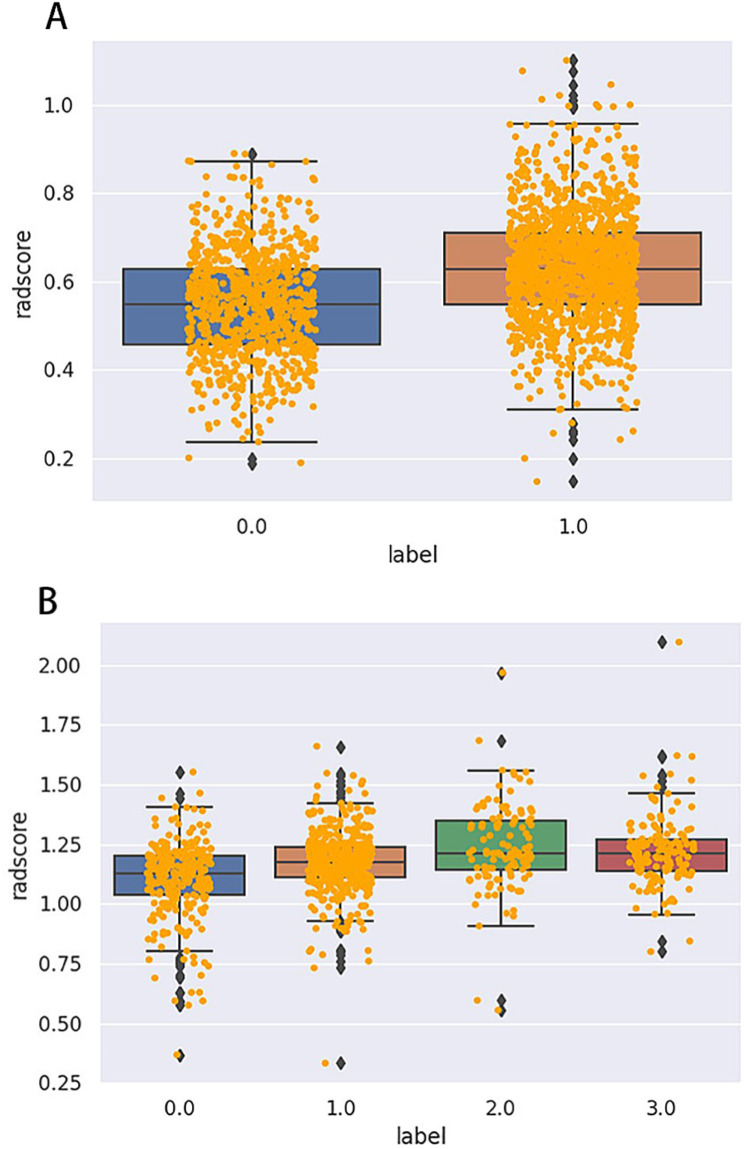
Radscores box plot of ultrasound image features for breast cancer postoperative recurrence risk (**A**) and molecular typing (**B**). The postoperative intermediate risk of recurrence was coded as 0, and the high risk of recurrence was coded as 1 (**A**). The HER-2 overexpression type was coded as 0, TNBC as 1, luminal A as 2, and luminal B as 3 (**B**). A total of 733 features were extracted from the ultrasound images of the patients. After normalizing the extracted features, we get Radscores. First, we find the upper edge, lower edge, median, and two quartiles of Radscores. Then, we connect the two quartiles to draw a box. Then, we connect the upper and lower edges to the box, and the median is in the middle of the box. The yellow dots represent the extracted features, and the blue diamonds represent outliers. In the figure, the median is in the middle of the box, and the data is normally distributed

**Fig. 2:**
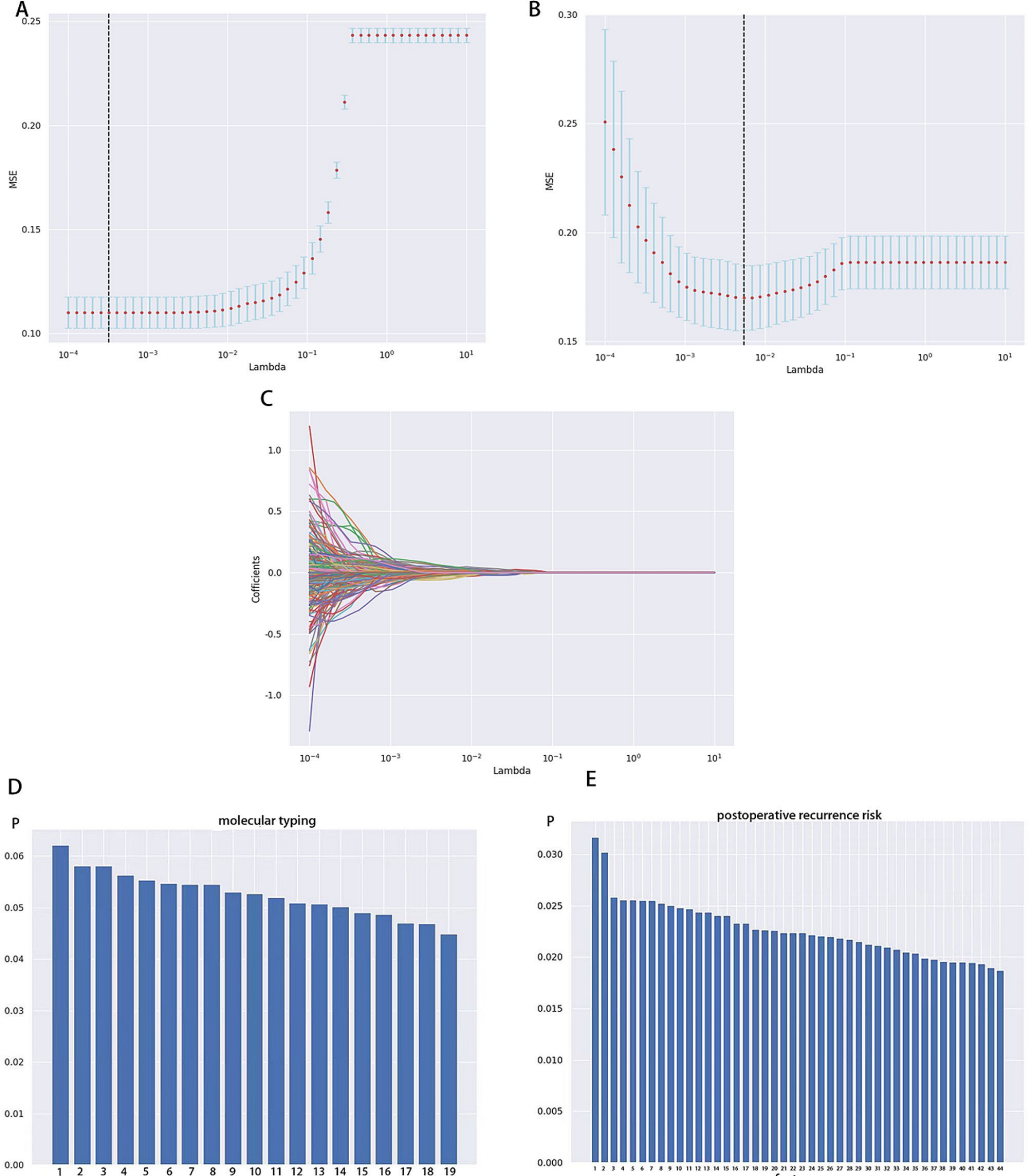
The independent samples t-test and LASSO regression were used to screen the significant features in molecular typing (**A**) and the risk of postoperative recurrence (**B**). In the process of the LASSO, the color line represents the coefficient of the feature with λ Value change curve, corresponding to dashed line λ Value is the best λ Value, keep the features where the coefficient is not 0 (**C**). Nineteen of the 733 features extracted from patient ultrasound images were associated with the risk of molecular typing (**D**, *P* < 0.05), the numbers represent the names of the optimal features in Table 5. Forty-four of the 733 features extracted from patient ultrasound images were associated with the risk of postoperative recurrence (**E**, *P* < 0.05), the numbers represent the names of the optimal features in Table 6. The bar plot shows p value for all the ultrasomic features used in the RadScore model in descending order of importance

Using the independent samples *t*-test and LASSO regression, the postoperative intermediate risk of recurrence was coded as 0, and the high risk of recurrence was coded as 1. In the other subgroup, the HER-2 overexpression type was coded as 0, TNBC as 1, luminal A as 2, and luminal B as 3. A total of 733 features were extracted from the ultrasound images of the patients, and features with an ICC greater than 0.75 were retained and weighted with the LASSO coefficient (Figs. 1 and 2A-C). Additionally, nineteen optimal features for the molecular typing of breast cancer were ultimately identified (refer to Table 5; Fig. 2D). A total of 44 optimal features for the risk of postoperative recurrence were identified (refer to Table 6; Fig. 2E). The radiomics models were subsequently constructed.

**Table 5:** Optimal characteristics for molecular typing

Optimal Characteristics	*P* value
log-sigma-2-mm-3D_glcm_ClusterShade	0.02
log-sigma-2-mm-3D_gldm_LargeDependenceEmphasis	< 0.01
log-sigma-2-mm-3D_glrlm_RunLengthNonUniformity	< 0.01
log-sigma-2-mm-3D_glszm_LargeAreaHighGrayLevelEmphasis	< 0.01
log-sigma-2-mm-3D_ngtdm_Busyness	0.01
original_glszm_GrayLevelNonUniformityNormalized	< 0.01
original_glszm_LargeAreaHighGrayLevelEmphasis	0.01
wavelet-HH_gldm_LargeDependenceEmphasis	< 0.01
wavelet-HL_firstorder_90Percentile	0.02
wavelet-HL_firstorder_Mean	< 0.01
wavelet-LH_firstorder_Median	0.02
wavelet-LH_glcm_Imc2	0.03
wavelet-LH_gldm_SmallDependenceLowGrayLevelEmphasis	< 0.01
wavelet-LH_glszm_SmallAreaEmphasis	< 0.01
wavelet-LH_ngtdm_Strength	< 0.01
wavelet-LL_gldm_LargeDependenceLowGrayLevelEmphasis	< 0.01
wavelet-LL_glszm_SmallAreaHighGrayLevelEmphasis	0.01
wavelet-LL_ngtdm_Complexity	< 0.01
wavelet-LL_ngtdm_Strength	< 0.01

Note: Nineteen of the 733 features extracted from patient ultrasound images were associated with molecular typing (*P* < 0.05). The names of the features are on the left, and the *P*-values from the LASSO analysis are on the right

**Table 6:** Optimal characteristics for the risk of postoperative recurrence

Optimal Characteristics	*P* value
original_glszm_ZoneVariance	< 0.01
original_glrlm_ShortRunEmphasis	< 0.01
original_glrlm_ShortRunLowGrayLevelEmphasis	< 0.01
original_ngtdm_Contrast	< 0.01
wavelet-LH_glcm_DifferenceAverage	< 0.01
wavelet-LH_glcm_Imc2	< 0.01
wavelet-LH_glszm_GrayLevelNonUniformity	< 0.01
wavelet-LH_glszm_ZoneEntropy	< 0.01
wavelet-LH_gldm_SmallDependenceLowGrayLevelEmphasis	< 0.01
wavelet-HL_firstorder_Mean	< 0.01
wavelet-HL_glcm_Imc1	0.01
wavelet-HL_glszm_LargeAreaHighGrayLevelEmphasis	< 0.01
wavelet-HL_glszm_ZoneEntropy	< 0.01
wavelet-HL_glrlm_RunLengthNonUniformity	0.03
wavelet-HH_firstorder_Energy	< 0.01
wavelet-HH_firstorder_Median	< 0.01
wavelet-HH_firstorder_TotalEnergy	< 0.01
wavelet-HH_glcm_Correlation	< 0.01
wavelet-HH_glcm_Imc1	< 0.01
wavelet-HH_glcm_Imc2	< 0.01
wavelet-HH_gldm_DependenceEntropy	< 0.01
wavelet-LL_glcm_ClusterShade	< 0.01
wavelet-LL_glcm_DifferenceVariance	< 0.01
wavelet-LL_glcm_InverseVariance	< 0.01
wavelet-LL_glcm_MaximumProbability	< 0.01
wavelet-LL_glszm_GrayLevelVariance	0.01
wavelet-LL_glszm_SmallAreaEmphasis	< 0.01
wavelet-LL_gldm_DependenceEntropy	< 0.01
wavelet-LL_gldm_DependenceNonUniformityNormalized	< 0.01
log-sigma-1-mm-3D_firstorder_Maximum	< 0.01
log-sigma-1-mm-3D_glcm_Imc1	0.03
log-sigma-1-mm-3D_glcm_MCC	0.02
log-sigma-1-mm-3D_glszm_LargeAreaLowGrayLevelEmphasis	< 0.01
log-sigma-1-mm-3D_glszm_SizeZoneNonUniformity	< 0.01
log-sigma-1-mm-3D_gldm_SmallDependenceLowGrayLevelEmphasis	< 0.01
log-sigma-2-mm-3D_firstorder_10Percentile	< 0.01
log-sigma-2-mm-3D_firstorder_Maximum	0.03
log-sigma-2-mm-3D_glcm_MCC	0.03
log-sigma-2-mm-3D_glrlm_RunEntropy	< 0.01
log-sigma-2-mm-3D_ngtdm_Busyness	< 0.01
log-sigma-3-mm-3D_firstorder_Maximum	< 0.01
log-sigma-3-mm-3D_firstorder_Mean	< 0.01
log-sigma-3-mm-3D_glcm_ClusterProminence	< 0.01
log-sigma-3-mm-3D_glcm_MaximumProbability	< 0.01

Note: Forty-four of the 733 features extracted from patient ultrasound images were associated with the risk of postoperative recurrence (*P* < 0.05). The names of the features are on the left, and the *P*-values from the LASSO analysis are on the right

#### Postoperative recurrence risk prediction model

**Fig. 3:**
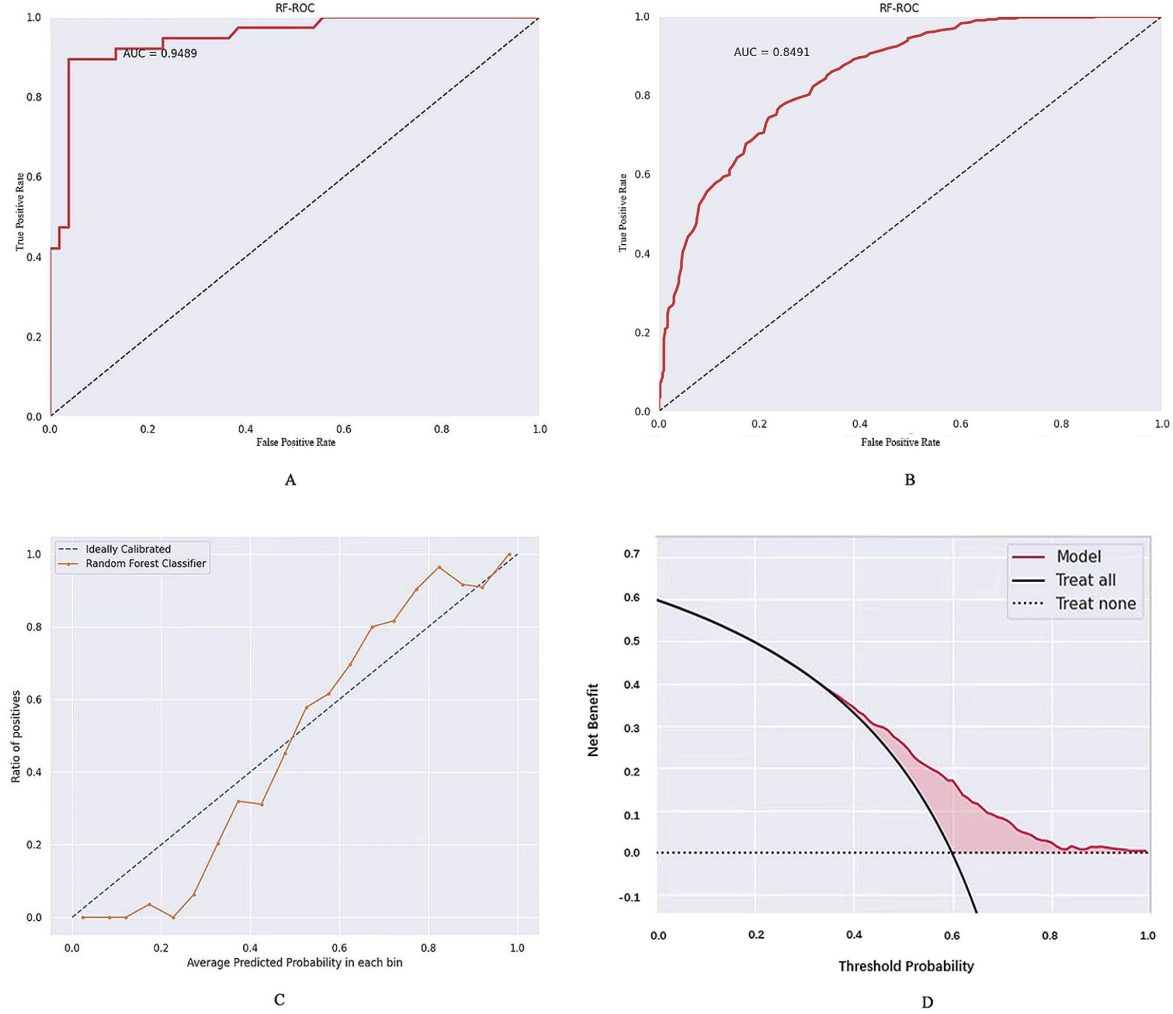
Predictive model for postoperative recurrence risk of breast cancer. **A**, the receiver operating characteristic (ROC) curves in training set. **B**, the ROC curves in validation set. **C**, calibration curves analysis of the predictive model. Diagonal dotted line indicates perfect prediction, while orange solid line indicates a model’s performance. Closer fitting to the diagonal dotted line indicates better performance. As shown in the figure, the model predicts good performance (*P* = 0.30). **D**, decision curves analysis of the predictive model. The red line represents the assumption that all patients have postoperative recurrence. The dotted line indicates the hypothesis that no patients have postoperative recurrence. Red shaded area represents the predictive effectiveness of the model

The AUC values for the postoperative recurrence risk prediction model constructed using ultrasound imaging omics features were 0.9489 and 0.8491 in the training set and the validation set, respectively (refer to Table 7; Fig. 3A and B). The calibration curve indicated that the ultrasound imaging omics model performed well in assessing the consistency of a particular result between the training and validation sets with the ideal model (refer to Fig. 3C, *P* = 0.30). Analysis of the decision curves demonstrated that clinical ultrasound imaging omics exhibited superior applicability in both the training and validation sets, showcasing enhanced diagnostic performance (refer to Fig. 3D).

**Table 7:** Performance evaluation of the postoperative recurrence risk models

	Training cohort			Validation cohort		
	AUC (95%CI)	Sen	Spe	AUC (95%CI)	Sen	Spe
postoperative recurrence risk	0.9489 (0.8263–0.9935)	0.8791	0.9158	0.8491 (0.7582–0.9257)	0.8534	0.8957

Note: AUC, area under the curve; 95% CI, 95% confdence interval; Sen, sensitivity; Spe, specificity

#### Molecular typing prediction model

**Fig. 4:**
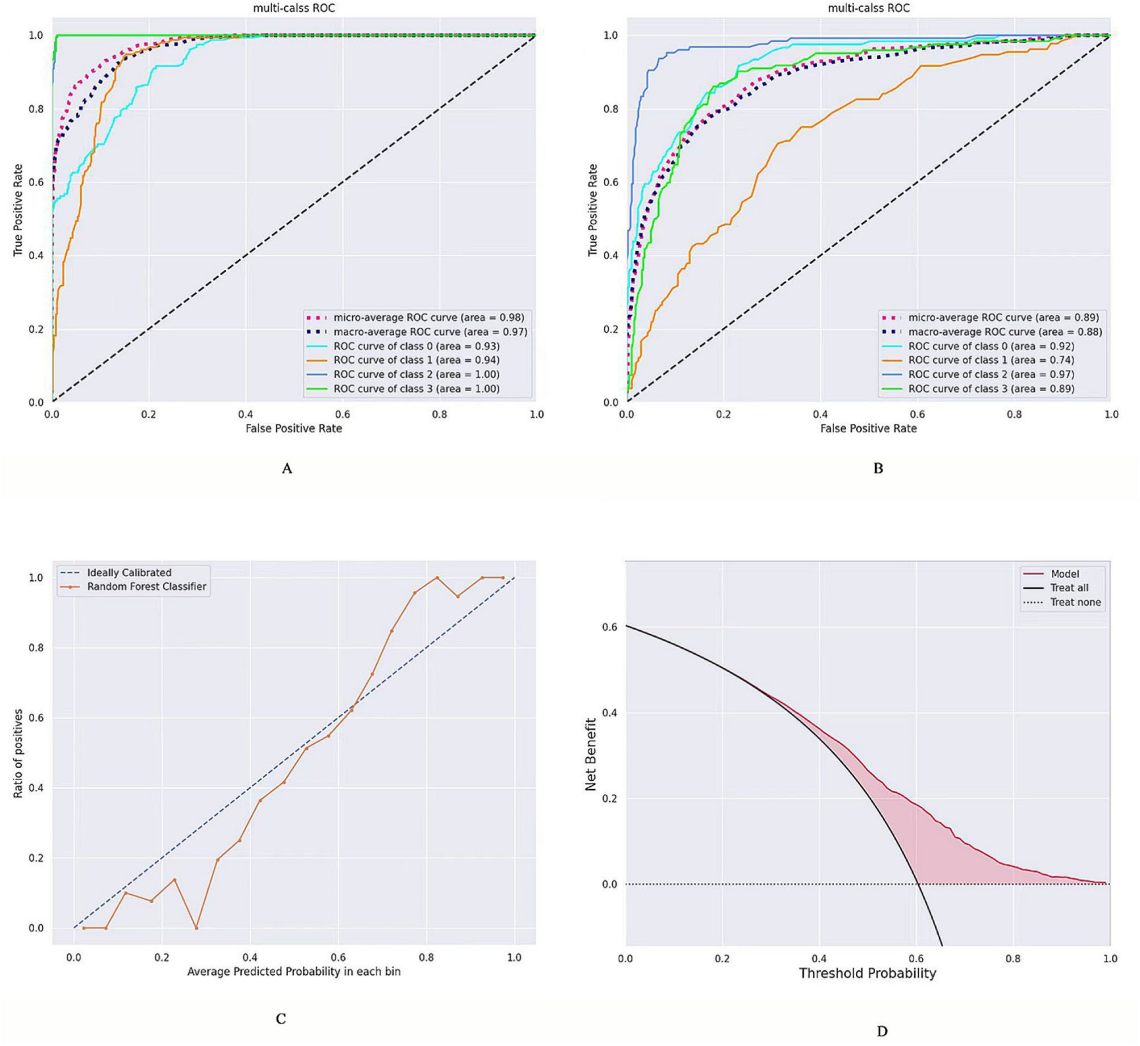
Predictive model for molecular subtyping of breast cancer. **A**, the receiver operating characteristic (ROC) curves in training set. **B**B, the ROC curves in validation set. **C**, calibration curves analysis of the predictive model. Diagonal dotted line indicates perfect prediction, while orange solid line indicates a model’s performance. Closer fitting to the diagonal dotted line indicates better performance. As shown in the figure, the model predicts good performance (*P* = 0.09). **D**, decision curves analysis of the predictive model. The red line indicates the hypothesis that all patients had different molecular types of breast cancer. The dotted line represents the hypothesis that none of the patients had different molecular types of breast cancer. The red shaded area indicates the predicted effect of the model

The corresponding AUC values for the molecular typing prediction model in the training set and validation set were as follows: 0.93 and 0.92 for the HER-2 overexpression phenotype, 0.94 and 0.74 for the TNBC phenotype, 1.00 and 0.97 for the luminal A phenotype, and 1.00 and 0.89 for the luminal B phenotype (refer to Table 8; Fig. 4A and B), respectively. The calibration curve indicated that the ultrasound imaging omics model performed effectively in assessing the consistency of a particular result between the training and validation sets with the ideal model (refer to Fig. 4C, *P* = 0.09). Analysis of the decision curves demonstrated that clinical ultrasound imaging omics exhibited enhanced applicability in both the training and validation sets, displaying superior diagnostic performance (refer to Fig. 4D).

**Table 8:** Performance evaluation of the molecular typing models

	Training cohort			Validation cohort		
	AUC (95%CI)	Sen	Spe	AUC (95%CI)	Sen	Spe
HER-2 overexpression	0.93 (0.83–0.96)	0.87	0.91	0.92 (0.80–0.98)	0.854	0.90
luminal A	0.94 (0.82–0.99)	0.81	0.90	0.74 (0.73–0.87)	0.72	0.83
luminal B	1.00 (0.86-1.00)	0.82	0.94	0.97 (0.85-1.00)	0.85	0.94
TNBC	1.00 (0.82-1.00)	0.86	0.96	0.89 (0.76–0.93)	0.88	0.0.91

Note: AUC, area under the curve; 95% CI, 95% confdence interval; Sen, sensitivity; Spe, specificity; TNBC, triple negative breast cancer

## Discussion

Recent studies indicate an increasing incidence of breast cancer, particularly affecting young adults. Assessing the risk of postoperative recurrence and molecular typing is crucial for making personalized treatment decisions and assessing prognosis in patients diagnosed with breast cancer. Currently, postoperative pathology and immunohistochemistry are common methods for assessing these risks. However, the challenge lies in rapidly performing these assessments through non-invasive means. High-frequency ultrasound is adept at clearly displaying the morphological characteristics of breast masses, and its non-invasive, rapid, and convenient nature has made it widely accepted as the preferred examination for breast cancer screening and assessing diagnostic and therapeutic efficacy in China [33]. In this study, we delved into the clinical characteristics of molecular typing and postoperative recurrence risk. Through univariate and logistic regression models, we discovered that predicting molecular typing and postoperative recurrence risk based solely on clinical characteristics proved to be ineffective. Consequently, we further explored the value of ultrasonography in predicting the molecular typing of breast cancer and the risk of postoperative recurrence. This exploration aims to provide evidence supporting the diagnosis and treatment of patients with breast cancer, facilitate timely adjustments in therapeutic direction, and assist in the clinical development of personalized treatment plans.

### Relationship between radiomics and clinical and imaging features with the risk of postoperative recurrence

Based on the postoperative recurrence risk assessment table in the CACA guidelines, patients were categorized into intermediate-risk and high-risk groups. Through univariate analysis and multifactorial logistic regression model analysis of the included clinical features, the number of lymph node metastases, ER expression, HER-2 expression, molecular typing, clinical staging, and ultrasonographic blood flow grading were identified as independent factors influencing the risk of postoperative recurrence. A total of 44 radiomic features were extracted and modeled, yielding AUC values of 0.9489 and 0.8491 for the postoperative recurrence risk prediction model in the training and validation sets, respectively. Notably, the radiomics model demonstrated superior predictive efficacy. This finding aligns with previous research, such as by Wang et al., who reported that a radiomics model assessing the risk of recurrence in patients with nasopharyngeal malignancies exhibited better predictive power than clinical, Ki-67-based, and TNM models [34]. Similarly, Qian et al. constructed a radiomics combined clinical model based on multiphase CT images and clinical risk factors, achieving AUCs of 0.813 and 0.838 in the training and validation sets, respectively [35]. This consistency supports the conclusion that radiomics outperforms clinical features in predicting the risk of cancer recurrence.

### The relationship between the radiomics and clinical and imaging features with molecular typing

Through univariate analysis and multifactorial logistic regression model analysis of the included clinical features, 6 independent risk factors were identified: Ki-67 expression, number of lymph node metastases, histological grading, risk of postoperative recurrence, clinical staging and lymph node morphology. Additionally, 19 radiomic features were extracted and modeled, resulting in respective AUC values for the molecular typing prediction model in the training set and validation set. Specifically, the AUC values were 0.93 and 0.92 for the HER-2 overexpression phenotype, 0.94 and 0.74 for the TNBC phenotype, 1.00 and 0.97 for the luminal A phenotype, and 1.00 and 0.89 for the luminal B phenotype. These results indicate that the ultrasound imaging omics model exhibited a strong predictive ability. In the results, both luminal A and luminal B types demonstrated an AUC of 1 in the training set, suggesting that the included radiomic features were relatively accurate, and there was no significant difference after feature extraction through multiple trainings. Consequently, the validation set performed well in this model. Notably, the ultrasound imaging omics model in this study outperformed previous research—Wu et al. achieved an overall accuracy of 74.1% in predicting 4 molecular types using X-ray, MRI, and clinical features, while Chen et al. attained a model AUC of 0.834 in distinguishing triple-negative breast cancer from non-triple-negative breast cancer using ultrasound imaging omics [36, 37]. The ultrasound imaging omics model in the current study demonstrated excellent performance, significantly enhancing the accuracy and robustness of predictions.

### Limitations

This study has certain limitations: (1) It is confined to a single center with a modest sample size, necessitating expansion in subsequent research endeavors to encompass a more extensive sample size and the implementation of diverse classification methodologies; (2) The ROI delineated are exclusively two-dimensional (2D), introducing susceptibility to the volume effect. Future investigations will address this limitation by delineating three-dimensional (3D) images; (3) The retrospective nature of this study, coupled with the subjective nature of ultrasound examinations and the static quality of the analyzed images, may result in the inadvertent omission of specific feature information; (4) Certain clinical features were subjected to semi-qualitative evaluation, introducing a degree of subjectivity.

## Conclusion

In conclusion, the model developed using ultrasound imaging omics features for breast cancer demonstrates robust diagnostic performance, effectively assessing the risk of postoperative recurrence, and exhibiting high accuracy and sensitivity in predicting the molecular typing of breast cancer. This offers clinicians more precise information for both diagnosis and treatment decisions. However, it is important to note that the usage of radiomics is currently in its early developmental stages, and its integration into the medical field will continue to evolve with the further advancement of data sharing and machine learning.

## Data Availability

The data that support the findings of this study are available from the corresponding author, upon reasonable request.

## Abbreviations

BC: Breast cancer
ROI: Region of interest
LASSO: Least absolute shrinkage and selection operator
SVM: Support vector machine
ROC: Receiver operating characteristic
AUC: Area under the curve
HER-2: Human epidermal growthfactor receptor 2
TNBC: Triple negative breast cancer
ER: Estrogen receptor
PR: Progestogen receptor
CSCO: Chinese society of clinical oncology
CACA: China anti-cancer association
MRI: Magnetic resonance imaging
NACT: Neoadjuvant chemotherapy
HR: Hormone receptors
GLCM: Gray-level co-occurrence matrix
GLRLM: Gray-level run-length matrix
GLSZM: Gray-level size zone matrix
GLDM: Gray level dependence matrix
ICC: Intra-class correlation coefficient
SMOTE: Synthetic minority oversampling technique
BI-RADS: Breast imaging reporting and data system
CT: Computed tomography
CI: Confidence interval
2D: Two-dimensional
3D: Three-dimensional
CDF: Icolor doppler flow imaging
SWE: Shear wave elastrography
