# Correction: Use of ultrasound imaging Omics in predicting molecular typing and assessing the risk of postoperative recurrence in breast cancer

**DOI:** 10.1186/s12905-024-03288-5

**Published:** 2024-08-14

**Authors:** Xinyu Song, Haoyi Xu, Xiaoli Wang, Wen Liu, Xiaoling Leng, Yue Hu, Zhimin Luo, Yanyan Chen, Chao Dong, Binlin Ma

**Affiliations:** 1 Department of Breast and Thyroid Surgery, Tumor Hospital Affiliated to Xinjiang Medical University, No. 789 of Suzhou Street, Xinshi District, Urumqi, 830000 China; 2 Department of Artificial Intelligence and Smart Mining Engineering Technology Center, Xinjiang Institute of Engineering, Urumqi, 830023 China; 3 Department of Ultrasound, The Tenth Affiliated Hospital of Southern Medical University, Dongguan, 523000 China; 4 Department of Breast Cancer Center Diagnosis Specialist, Sun Yat-sen Memorial Hospital, Guangzhou, 510120 China; 5 Department of General Surgery, Tori County People’s Hospital, Tuoli, 834500 China

**Correction: BMC Women’s Health (2024) 24:380**

10.1186/s12905-024-03231-8

Following publication of the original article [1], in the Funding Section, the grant number relating to Department of Science and Technology of Xinjiang Uygur Autonomous Region was incorrectly given as 2022TSYCTD0001 and should have been 2022TSYCTD0017.

The original article has been corrected.
